# Intracellular protein crystallization in living insect cells

**DOI:** 10.1002/2211-5463.70020

**Published:** 2025-03-28

**Authors:** Robert Schönherr, Nina Eichler, Fatama A. Sornaly, Juliane Boger, Anne M. Frevert, Janine Mia Lahey‐Rudolph, Hannah Meyer, Lisa Weymar, Lars Redecke

**Affiliations:** ^1^ Institute of Biochemistry University of Lübeck Germany; ^2^ Center for Free‐Electron Laser Science (CFEL) Hamburg Germany; ^3^ Photon Science, Deutsches Elektronen Synchrotron (DESY) Hamburg Germany; ^4^ Present address: Department of Applied Natural Sciences, TH Lübeck University of Applied Sciences Lübeck Germany

**Keywords:** baculovirus, *in cellulo* crystallization, InCellCryst, protein crystallization, serial X‐ray diffraction, X‐ray crystallography

## Abstract

Crystallization of recombinant proteins in living cells is an emerging approach complementing conventional crystallization techniques. Homogeneous microcrystals well suited for serial diffraction experiments at X‐ray free‐electron lasers and synchrotron sources can be produced in a quasi‐native environment, without the need for target protein purification. Several protein structures have already been solved; however, exploiting the full potential of this approach requires a systematic and versatile screening strategy for intracellular crystal growth. Recently, we published InCellCryst, a streamlined pipeline for producing microcrystals within living insect cells. Here, we present the detailed protocol, including optimized target gene expression using a baculovirus vector system, crystal formation, detection, and serial X‐ray diffraction directly in the cells. The specific environment within the different cellular compartments acts as a screening parameter to maximize the probability of crystal growth. If successful, diffraction data can be collected 24 days after the start of target gene cloning.

## Graphical abstract

This research protocol provides a step‐by‐step guide for applying the InCellCryst pipeline for intracellular protein crystallization. After gene cloning and generation of recombinant baculoviruses, High Five insect cells are infected for target protein crystallization. If detection is successful, the crystal‐containing cells are mounted for serial X‐ray diffraction data collection directly in the cells, followed by data processing and structure elucidation.

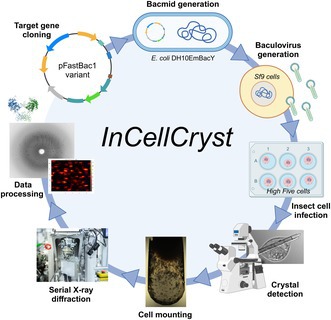

**Abbreviations**
a/a: antibiotic‐antimycotic
AP: alkaline phosphatase
bp: base pair
DIC: differential interference contrast
DNA: deoxyribonucleic acid
*E. coli*
: *Escherichia coli*
EYFP: enhanced yellow fluorescent protein
FD: fast digest
fwd: forward
kb: kilobase
MOI: multiplicity of infection
N_2_
: nitrogen
PCR: polymerase chain reaction
PEG: polyethylene glycol
pFB1: pFastBac1
rBV: recombinant baculovirus
rev: reverse
RT: room temperature
SAXS: small‐angle X‐ray scattering
SOB: super optimal broth
TAE: tris‐acetate‐EDTA
TCID_50_
: 50% tissue culture infection dose
TEM: transmission electron microscopy
XRPD: X‐ray powder diffraction
YT: yeast tryptone

The crystalline state of a protein is the prerequisite to obtaining structural information at atomic resolution by applying X‐ray crystallography. It is nowadays well established that living cells from all kingdoms of life can form intracellular protein crystals, denoted as ‘*in cellulo* crystals’ [1]. The advent of high‐brilliance synchrotron sources, X‐ray free‐electron lasers, and improved serial data collection strategies [2, 3, 4, 5] has allowed the use of these micrometer‐sized crystals for structural biology. Today, a significant number of *in cellulo* crystals, grown in native environments or in a host cell as a consequence of recombinant protein production, have been used to elucidate protein structures [6, 7, 8, 9, 10, 11, 12, 13, 14, 15, 16, 17, 18, 19, 20, 21, 22, 23, 24, 25]. Also because of the availability of the native cofactors inside the living cell, intracellular protein crystallization in a quasi‐native environment offers exciting new possibilities for X‐ray crystallography, complementing conventional crystallization approaches.

To better exploit the cellular crystallization capabilities and broaden the user community, we recently reported InCellCryst, an advanced and streamlined approach for the generation and detection of intracellular crystals in insect cells that allows, at best, collecting serial X‐ray diffraction data for the structure elucidation of the crystallized protein within 24 days [26]. The hallmarks of this pipeline include a highly versatile cloning system, the possibility to use the differing chemical environments in cellular compartments as suitable screening parameters to maximize the chance for target protein crystallization, serial diffraction data collection directly in viable insect cells without the need for crystal isolation, and finally, state‐of‐the‐art data processing.

In this research protocol, we now provide a detailed, step‐by‐step guide for improving the probability of obtaining intracellular crystals from proteins recombinantly produced in insect cells. The PCR‐amplified target gene is cloned into a pFastBac1 (pFB1) plasmid (Bac‐to‐Bac system, Invitrogen, ThermoFisher Scientific, Waltham, MA, USA) using a ligation‐based approach and cohesive ends (Fig. 1A). Since crystallization of recombinant proteins occurs in different insect cell compartments [18, 27, 28, 29, 30], we constructed a library of pFB1 plasmids that encode different cellular localization sequences, together with start‐ and stop‐codons (Fig. 2) [26]. This directs the target protein into different chemical environments for crystallization screening. After cloning, parts of the recombinant pFB1 plasmids are transposed into the baculoviral genome using Tn7 transposition in *E. coli* DH10EmBacY cells [31], followed by recombinant bacmid isolation and PCR validation of the correct transposition (Fig. 1B). The bacmid is the engineered, circular backbone of the baculoviral genome, constituting a Kanamycin resistance to enable antibiotic selection and an EYFP fluorescence gene for direct monitoring of cell infection and easy determination of viral titers. Recombinant baculoviruses (rBVs) are produced by the transfection of Sf9 insect cells and subsequent virus passaging, until virus titration using an endpoint dilution assay [26] on High Five insect cells reveals a sufficiently high titer (Fig. 1C). For intracellular crystallization screening, transient rBV infection of High Five cells at an MOI of 1 was usually optimal to obtain the largest crystal sizes and highest percentage of crystal‐containing cells in the culture between 72 and 96 h post‐infection (hpi) [26] (Fig. 1D). A strong indication of successful protein crystallization is the detection of highly ordered structures with sharp edges in a cell, which can be most conveniently performed using light microscopy with a high NA objective in combination with differential interference contrast (DIC). However, proof of crystallinity can only be established by the diffraction of X‐rays, e.g., using the SAXS‐XRPD approach [32], by second harmonic generation (SHG) imaging [33], or by visualization of the crystal lattice, e.g., by transmission electron microscopy (TEM) [7, 18, 26, 28]. For data collection from intact crystal‐containing cells at 100 K at a synchrotron source, the cells are carefully transferred onto a MicroMesh mounted on a light microscope stage, covered with 40% PEG200 for cryoprotection, and manually cryo‐cooled in liquid nitrogen (Fig. 1E). Serial diffraction data of the crystals contained in viable cells can be collected using a helical grid scan approach [23, 26, 34], which is, e.g., available at the EMBL P14 synchrotron beamline located at the PETRA III storage ring (DESY, Hamburg, Germany). A full list of X‐ray‐based facilities where data from microcrystals can be collected is presented in [35]. For data processing and structure elucidation, which is not part of the protocol presented here, the software suite crystfel, which is optimized for still images in high multiplicity [36] is recommended.

**Fig. 1:**
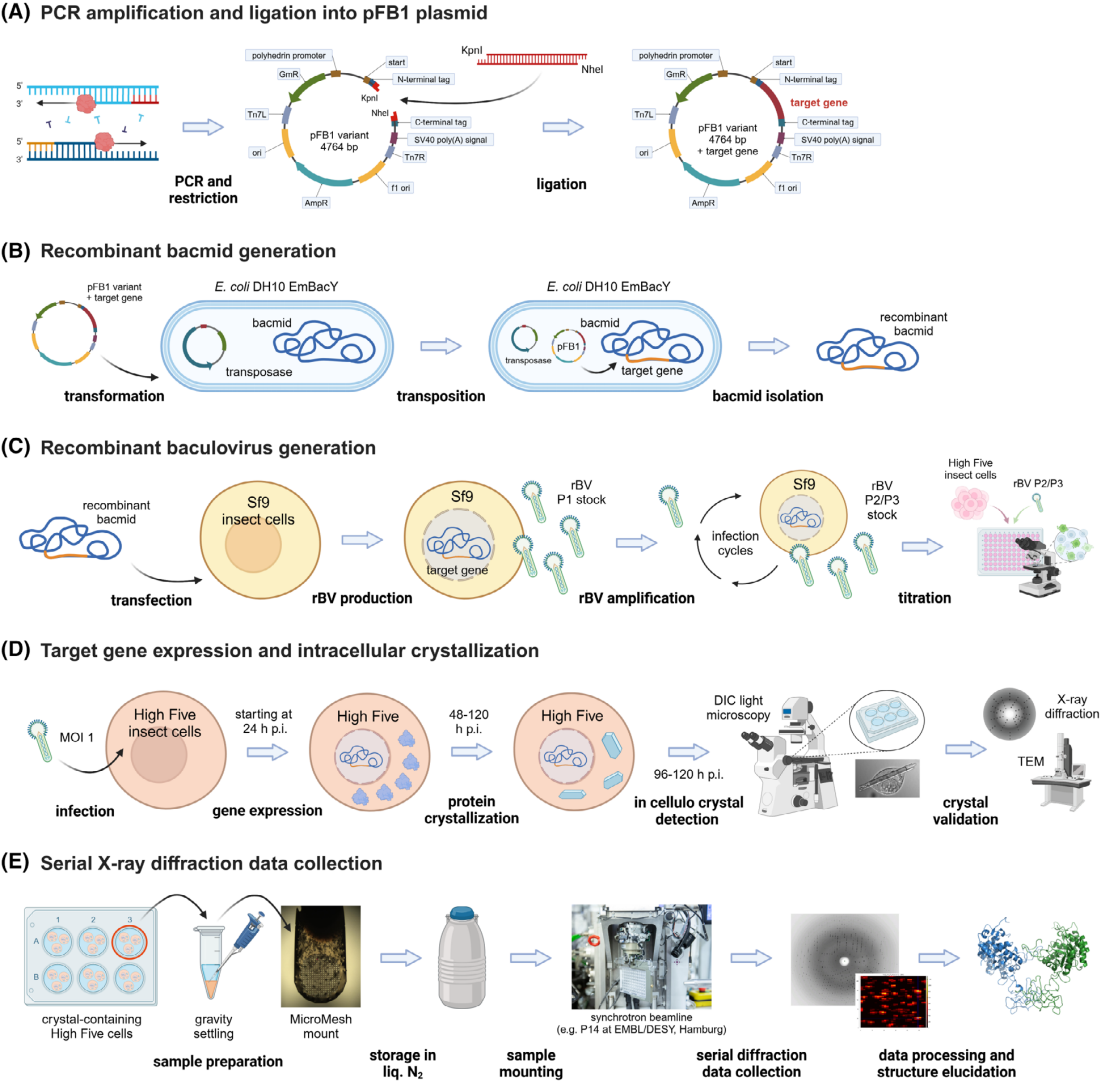
Schematic summary of the InCellCryst pipeline. (A) The gene of interest is amplified by PCR and ligated into modified pFastBac1 plasmids. (B) After the transformation of *E. coli* DH10EmBacY cells, recombination with the bacmid takes place. The recombinant bacmid is isolated and (C) Sf9 insect cells are transfected for rBV generation. After high titer viral stock production, (D) High five insect cells are infected and used for high‐yield target gene expression. This eventually leads to the crystallization of the target protein within one of the cellular compartments, depending on the transport signal fused to the target protein sequence. (E) Crystal‐containing cells are directly used for serial diffraction data collection at 100 K at a synchrotron source. Serial diffraction data is finally processed using CrystFEL to elucidate the structure of the target protein. p.i., post‐infection; rBVs, recombinant baculoviruses. Created with BioRender.com.

**Fig. 2:**
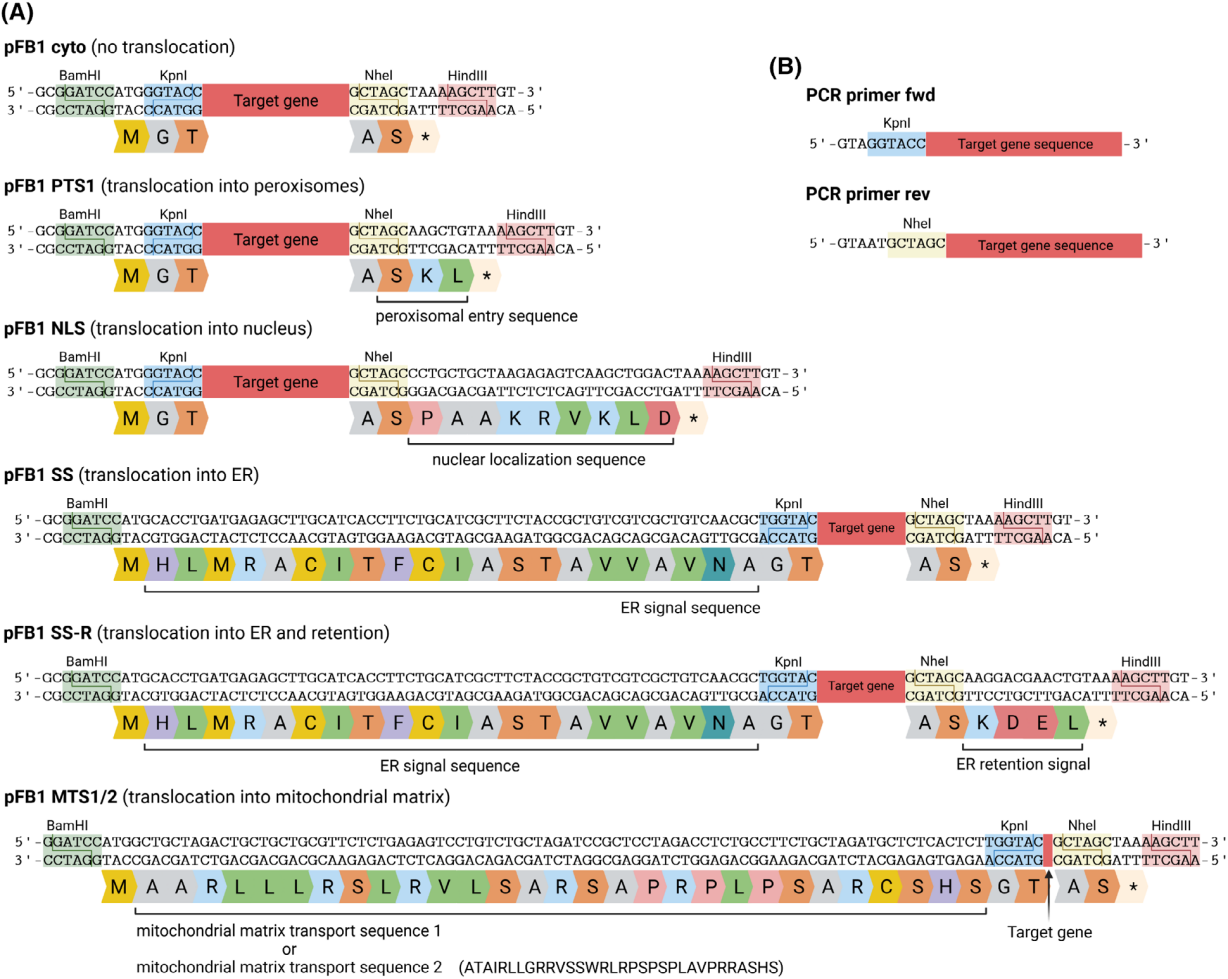
Target gene cloning. (A) Schematic representation of modified pFB1 plasmids encoding cellular translocation sequences to transport the target protein co‐ or post‐translationally into cellular compartments that are characterized by individual environmental conditions. Cleavage with *Kpn*I and *Nhe*I allows target gene cloning in frame with the start/stop codon and the translocation sequences encoded in the plasmids. Cleavage with *Bam*HI and *Hind*III removes the entire cloning cassette from the plasmid. These pFB1 plasmids are also available encoding an additional HA tag (YPYDVPDYA) upstream of the *Kpn*I site for N‐terminal fusion or downstream of the *Nhe*I site for C‐terminal fusion to the target protein. Moreover, a pFB1 cyto N‐His plasmid was designed to encode a His tag, an HA tag, and a TEV protease cleavage site upstream of the *Kpn*I site. (B) Example of PCR primers for target gene amplification. At the 5′ ends, the fwd and rev primers need to encode a *Kpn*I and *Nhe*I restriction site, respectively. Moreover, random nucleotides should be added at the ends to improve the efficiency of the PCR product cleavage. Created with BioRender.com. Full sequences of the presented pFB1 plasmids are provided as Supporting Information.

As a supplement to conventional methods, InCellCryst opens a quick and easy route to increase the success rate of intracellular crystallization, particularly by the compartment screening option. Ordered structures indicating a successful in cellulo crystallization have been observed for a diverse variety of proteins (Fig. 3) [1, 7, 15, 18, 23, 26, 28, 30, 32]. However, strongly depending on the target protein, the fraction of crystal‐containing cells within a culture varies between more than 90% and less than 1%, representing a bottleneck of this approach, if successful at all. As a rule of thumb, 10% of cells should contain a crystal to enable the serial collection of a full data set, already resulting in the structure elucidation of several proteins [7, 15, 18, 23, 26, 28, 30]. Moreover, the rich source of biomolecules in the cellular environment allowed the identification of native ligands by co‐crystallization at quasi‐physiological conditions [18, 26], representing a unique feature of InCellCryst. If the screen of the different cellular compartments does not produce crystals or produces crystals with low efficiency or insufficient diffraction quality, variation of the insect cell line used for crystallization (e.g., Sf9 cells instead of High Five cells) or stepwise increase of the MOI up to 20 has been most promising for optimization trials so far. The addition of fetal bovine serum (FBS) can also positively affect the recombinant protein production in the cells [26], while changes in the cultivation temperature did not have any benefit. Moreover, the addition of chemicals, e.g., brefeldin A that induces ER stress, to the cell culture medium during crystal growth was reported [37] but the effect on intracellular protein crystallization needs to be systematically evaluated.

**Fig. 3:**
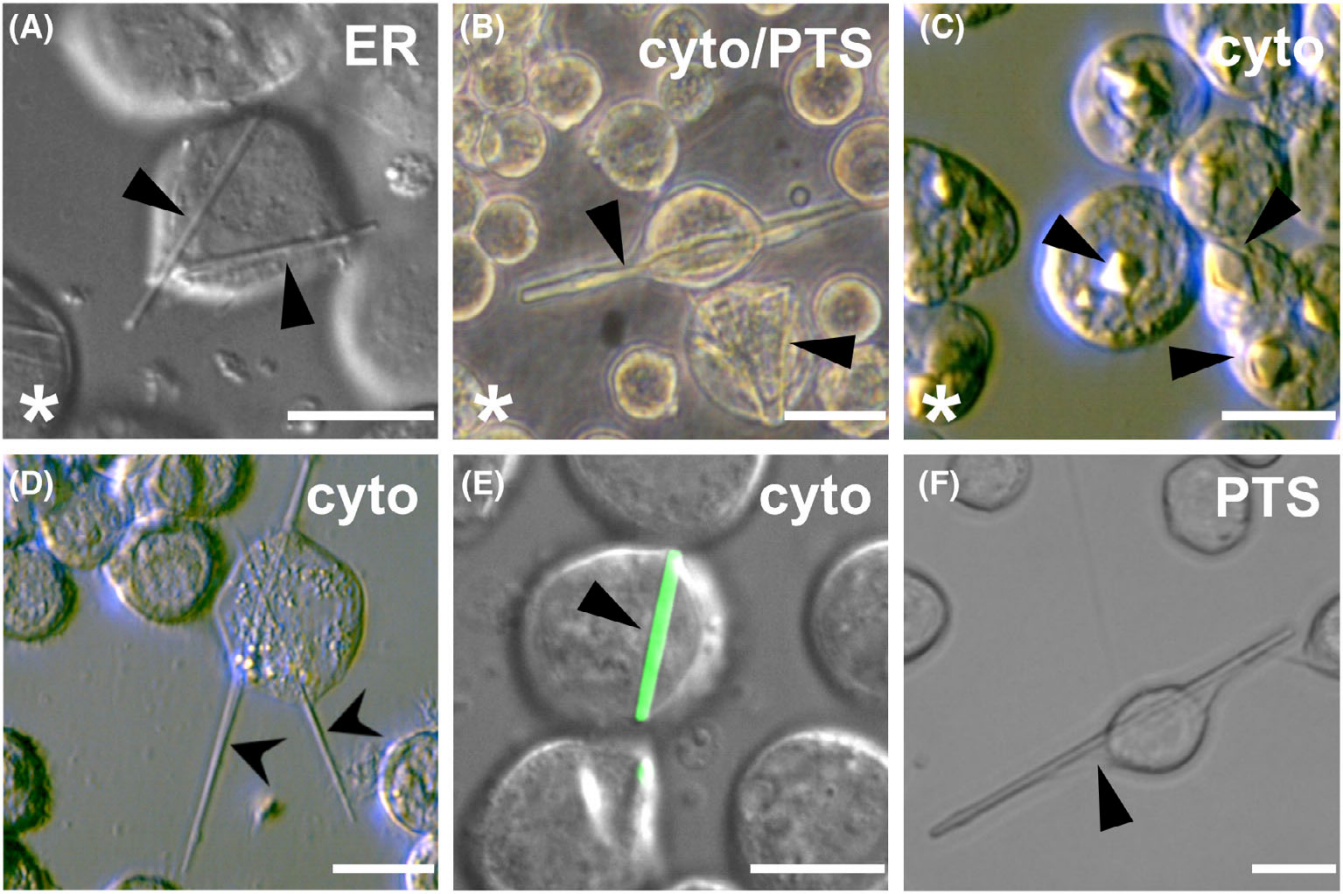
High Five insect cells containing ordered structures. Cells were infected with rBVs encoding (A) *Trypanosoma brucei* cathepsin B (CatB) [7, 23], (B) *Trypanosoma brucei* inosine monophosphate dehydrogenase (IMPDH) [18, 27], (C) *Neurospora crassa* HEX‐1 [15, 27], (D) Zika virus NS5 methyltransferase, (E) μNS protein from avian reovirus N‐terminally fused to GFP [29], and (F) *Photinus pyralis* luciferase [29] at an MOI of 1. Imaging followed 4 days post infection (dpi) i on a Nikon Ti2‐E or Ts2R‐FL microscope equipped with 100× objectives using the differential interference contrast (DIC) mode. Regular objects with sharp edges and mostly needle‐shaped morphology were detected (arrows). The translocation signal addressing a specific cellular compartment is indicated (cyto, cytosol; ER, endoplasmic reticulum; NLS, nucleus; PTS, peroxisomes). *, protein structure has already been elucidated using intracellularly grown crystals. Size bars for all images represent 20 μm.

## Materials

**Fig. 4:**
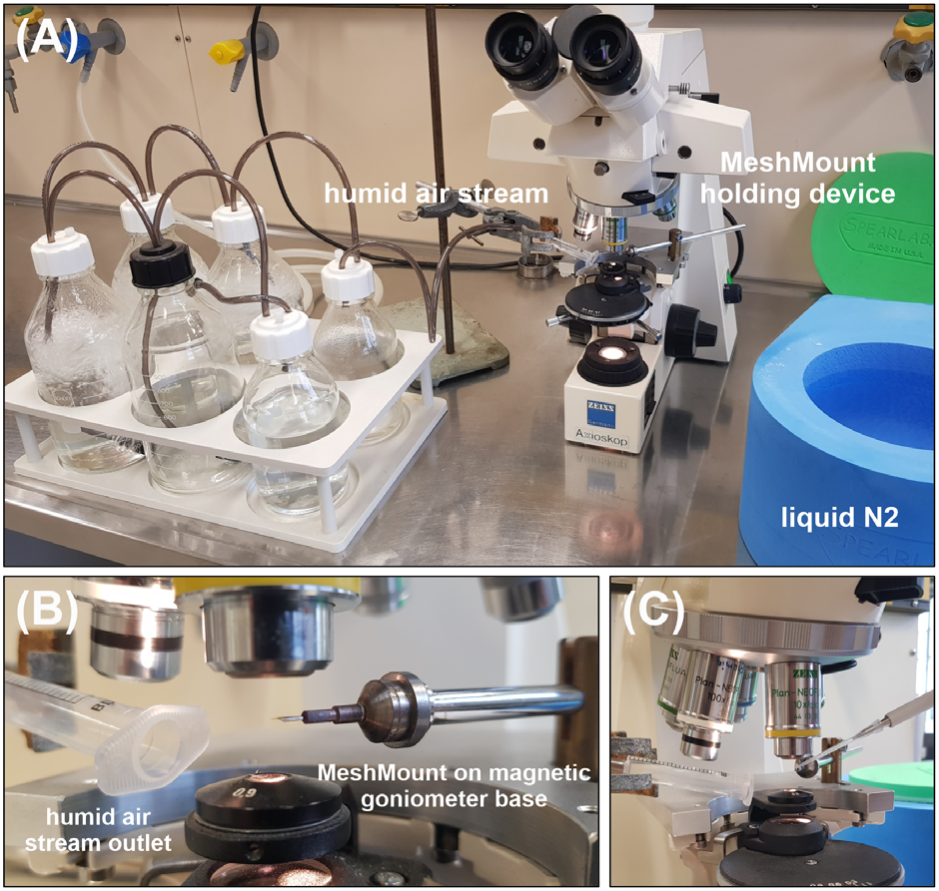
Laboratory setup for MicroMesh loading. (A) A standard upright cell culture light microscope is equipped with a custom‐made holding device for a MicroMesh mounted on a magnetic goniometer base. A continuous humid air stream to keep the cells hydrated during loading is produced by passing compressed air through different water‐filled bottles (left). An empty bottle at the end prevents sample contamination with water droplets. (B) and (C) After adjusting the MeshMount in the optical focus of the microscope, the cells are directly pipetted onto the surface, followed by the removal of the excess liquid using an extra fine liquid wick from the back of the mesh. Immediately after applying cryo protection by pipetting 40% PEG200 on the loaded cells and blotting of exceeding liquids, the MeshMount is flash‐frozen in liquid N_2_.

1. pFB1 plasmids (Invitrogen) modified by the insertion of intracellular protein translocation signals and fusion tags [26] (Fig. 2).
2. ALLin™ HiFi DNA polymerase (HighQu #HLE0201, Kraichtal, Germany).
3. Target gene‐specific PCR primers.
4. Agarose NEEO Ultra Quality (Carl Roth #2267.1, Karlsruhe, Germany).
5. TAE buffer (pH 8.3): 40 mm Tris, 1 mm EDTA, 10 mm acetic acid.
6. FastDigest *Kpn*I, *Nhe*I, *Bam*HI, and *Hind*III restriction enzymes and FastAP (Thermo Scientific).
7. T4 DNA Ligase (Thermo Scientific, Waltham, MA, USA).
8. Chemically competent *E. coli* DH5α cells (Invitrogen #18265017) or an equivalent strain.
9. Chemically competent *E. coli* DH10EmBacY cells (Geneva Biotech, Geneva, Switzerland).
10. SOB medium: 31 g·L^−1^ YT (Carl Roth), 20 mm MgCl_2_, 20 mm MgSO_4_.
11. YT Amp agar plates: 20 g·L^−1^ agar, 31 g·L^−1^ YT, 100 μg·mL^−1^ ampicillin (Gerbu #1046, Heidelberg, Germany).
12. YT Bac agar plate: 20 g·L^−1^ agar, 31 g·L^−1^ YT, 50 μg·mL^−1^ kanamycin (Gerbu #1291, Heidelberg, Germany), 7 μg·mL^−1^ gentamicin (Gerbu #1090), 10 μg·mL^−1^ tetracycline (Serva #35866, Heidelberg, Germany), 100 μg·mL^−1^ X‐Gal (Sigma Aldrich #6930‐OP), 40 μg·mL^−1^ IPTG (Sigma Aldrich #16758, St. Louis, MO, USA).
13. NucleoSpin® Gel and PCR Clean‐up Kit (Macherey‐Nagel #740609, Düren, Germany)
14. NucleoSpin® Plasmid Kit (Macherey‐Nagel #740588)
15. ZR BAC DNA Miniprep Kit (Zymo Research #D4049, Freiburg, Germany)
16. Primer pFB1 Seq fwd – 5′‐GTTGGCTACGTATACTCCGGA‐3′
17. Primer pFB1 Seq rev – 5′‐TTCAGGTTCAGGGGGAGGTG‐3′
18. Primer pUC/M13 fwd – 5′‐CCCAGTCACGACGTTGTAAAACG‐3′
19. Primer pUC/M13 rev – 5′‐AGCGGATAACAATTTCACACAGG‐3′
20. *Spodoptera frugiperda* Sf9 insect cells (Invitrogen #B82501 or Merck #71104, Darmstadt, Germany).
21. High Five insect cells (*Trichoplusia ni*, BTI‐Tn‐5B1‐4, Thermo Scientific #B85502).
22. Serum‐free ESF921 insect cell culture medium (Expression Systems #96‐001‐01, Davies, CA, USA).
23. Antibiotic‐Antimycotic (a/a, 100×, Gibco #15240062, ThermoFisher Scientific, Waltham, MA, USA).
24. ESCORT IV transfection reagent (Sigma Aldrich #L3287).
25. Sterile T25 (25 cm^2^) and T75 (75 cm^2^) angled cell culture flasks with non‐treated surfaces and filter caps (Sarstedt #83.3910.002 and #83.3911.002, Nümbrecht, Germany).
26. Sterile serological pipettes with cotton plug, pipette aid, and sterile 50‐mL conical tubes.
27. Sterile 6, 12, and 96 multi‐well plates (Greiner Bio‐One #657160, #665180 and #650161, Frickenhausen, Germany).
28. Sterile 2 mL aerosol‐tight tubes (Biozym #710768, Hessisch Oldendorf, Germany).
29. Sterile glass coverslip, round, 25 mm in diameter, No. 1 (VWR #631‐0171).
30. MicroMesh 700/25 mount (MiTeGen #M3‐L18SP‐25L, Ithaca, NY, USA)
31. Magnetic reusable goniometer base type B5 (MiTeGen #GB‐B5‐R).
32. Extra fine liquid wicks (MiTeGen #W‐XF).
33. Air humidifying device (Fig. 4).
34. 40% PEG200 in ESF921 medium (Sigma Aldrich #8.07483).
35. Liquid nitrogen.
36. PCR thermocycler, refrigerated centrifuge, spectrophotometer, laboratory shaker, plate incubator, incubation shaker, cell incubator with thermoregulation, orbital shaker, tissue culture hood, cell counter, cell culture light microscope, light microscope with differential interference contrast (DIC), fluorescence microscope with EYFP filter set, multi‐channel pipette, customized coverslip holder, immersion oil.

## Methods

### Cloning of target genes into modified pFB1 vectors

1. Amplify the target gene by PCR using gene‐specific primers (Tips & Tricks 1). PCR program (35 cycles of step 2–4):

**Table:** 

Initial denaturation	95 °C	2 min
Denaturation	95 °C	15 s
Annealing	Calculates	15 s (Tips & Tricks 2)
Elongation	72 °C	20 s·kb^−1^
Final elongation	72 °C	2.5× elongation time

2 Purify the PCR products by agarose gel electrophoresis (1% agarose with 2 μL per 100 mL Midori Green Xtra (NIPPON Genetics, Düren, Germany) in 1× TAE buffer), excise the desired DNA bands under blue light excitation, and extract the DNA following the Gel and PCR Clean‐up Kit's guidelines (Tips & Tricks 3).
3 Determine the DNA concentration and purity by absorption measurement at 260/280 nm (e.g., using a Nanodrop spectrophotometer).
4 Digest the purified PCR products and 2 μg of the pFB1 plasmid (Tips & Tricks 4) with FD *Kpn*I and FD *Nhe*I for 45 min at 37 °C. Add FastAP to dephosphorylate the plasmid DNA. Inactivate the enzymes by incubation at 80 °C for 5 min.
5 Purify the digested plasmid and PCR products by agarose gel electrophoresis and subsequent DNA gel extraction (step 2).
6 For ligation, calculate the appropriate amount of insert to achieve a molar ratio of at least 3 : 1 (insert : vector) using 50 ng plasmid. Add T4 DNA ligase and incubate for 1 h at RT. Prepare a negative control without the insert.
7 Thaw 20 μL of chemically competent *E. coli* DH5α (or equivalent strain) on ice. Add 2 μL of the ligation reaction to the cells and mix gently by flicking the tube (Tips & Tricks 5). Incubate on ice for at least 20 min, then heat shock the cells for 30 s at 42 °C. Incubate on ice for 2 min before adding 200 μL of SOB medium without antibiotics. Incubate at 37 °C for 1 h. Plate 80 μL of the cell suspension on a YT Amp agar plate. Concentrate the leftovers by centrifugation for 5 min at 1000 ***g***, remove 60 μL of the supernatant, resuspend the cells, and plate the remaining 80 μL. Incubate the plates at 37 °C for 16–20 h.
8 Compare the colony growth on the negative control plate to the plates with insert‐containing ligations. If the latter shows significantly more colonies, pick two colonies to inoculate in 7 mL of SOB medium containing 100 μg·mL^−1^ ampicillin. Incubate with shaking (270 r.p.m.) at 37 °C for 16–20 h.
9 Pellet the cells and purify the plasmid DNA following the Plasmid DNA Kit's guidelines.
10 Confirm successful ligation by digesting 1 μg of plasmid DNA with 1 μL FD *Bam*HI and 1 μL FD *Hind*III in a 20 μL reaction volume for 45 min at 37 °C. Perform agarose gel electrophoresis to visualize the DNA bands (step 2) (Tips & Tricks 6).
11 Determine the plasmid concentration and purity (step 3). Confirm mutation‐free plasmids by DNA sequencing using the primers pFB1 Seq fwd and pFB1 Seq rev.

### Generation of recombinant bacmid DNA

1. Thaw 20 μL of chemically competent *E. coli* DH10EmBacY cells on ice.
2. Add 40 ng pFB1 plasmid encoding the target protein to the cells and gently mix by flicking the tube. Incubate on ice for 20 to 30 min, then heat shock the cells at 42 °C for 30 s. Immediately incubate the suspension on ice for 2 min. Add 380 μL of SOB medium without antibiotics and incubate at 37 °C for 6 h at 500 r.p.m. Plate 100 μL of the cell suspension on a YT Bac agar plate. Concentrate the leftovers by centrifugation for 5 min at 1000 ***g***, remove 200 μL of the supernatant, resuspend the cells, and plate the remaining 100 μL. Incubate the plates at 37 °C for 2 days.
3. Check the plates for blue and white colonies. Pick two white colonies per plate and streak them onto a new YT Bac agar plate to isolate mixed clones. Incubate at 37 °C for an additional 2 days.
4. Pick two white colonies per plate to inoculate 7 mL of SOB medium containing 50 μg·mL^−1^ kanamycin and 7 μg·mL^−1^ gentamicin. Incubate with shaking (250 r.p.m.) at 37 °C for 16–20 h.
5. Isolate the bacmid DNA following the ZR BAC DNA Miniprep kit's guidelines. Elute the DNA with 30 μL of prewarmed (70 °C) elution buffer.
6. Confirm the transposition of the target gene by PCR (30 cycles) using the pUC/M13 fwd and rev primers (66 °C annealing temperature) and 10 ng bacmid DNA as a template. Use an elongation time of 30 s·kb^−1^ of the expected PCR product, which should have a length of 2300 bp plus the target gene size if transposition is successful. Unsuccessful transposition is indicated by a 300 bp DNA band. Perform agarose gel electrophoresis for evaluation (see above).

### Thawing and culturing of Sf9 and High Five insect cells

1. Thaw one vial of 5 × 10^7^ cryopreserved cells in a 37 °C water bath, while gently turning and moving the tube, until the ice in the vial is just fully thawed (Tips & Tricks 7).
2. Transfer the entire cell suspension (1 mL) into a tube containing 39 mL of prewarmed ESF921 medium (+a/a, 27 °C) and gently invert the tube to fully resuspend the cells. Centrifuge the cells at 100 ***g*** for 5 min to remove DMSO from the cell suspension. Resuspend the pellet in fresh 40 mL of prewarmed ESF921 (+a/a) medium.
3. Transfer half of the cell suspension each to a 75 cm^2^ cell culture flask. Incubate the flask upright on an orbital shaker in a cell incubator at 27 °C and 100 r.p.m. for 2 to 3 days. If necessary, increase the orbital shaker speed until a homogeneous cell suspension is established (max. 120 r.p.m.).
4. Count cells to calculate cell density and cell viability, if necessary. The first subculture should be performed when the viable cell density reaches 4 × 10^6^ cells·mL^−1^.
5. Subculturing: For the first subculture, dilute the cells in prewarmed ESF921 medium (+a/a) in a 75 cm^2^ culture flask. Adjust the cell density to 1 × 10^6^ cells·mL^−1^ in 20 mL culture volume. For subsequent subcultures, an initial cell density of 0.5 to 1 × 10^6^ cells·mL^−1^ can be used. Incubate for 2–3 days and perform the next subculture when the cell density exceeds 2 or 6 × 10^6^ cells·mL^−1^ for High Five and Sf9 cells, respectively (Tips & Tricks 8).
6. Media change: Transfer the cell suspension to a 50 mL conical tube and centrifuge at 100 ***g*** for 5 min. Discard the supernatant and resuspend the cell pellet in 20 mL prewarmed fresh ESF921 medium (+a/a). Incubate in a 75 cm^2^ culture flask at standard conditions for 2–3 days.

### Generation of recombinant baculovirus and virus stock titration

1. Plate 0.5 × 10^6^ Sf9 cells in 0.5 mL ESF921 medium (−a/a) per well in a 12‐well plate to facilitate adherence to the well surface and improve confluency. Allow the cells to adhere for 1 h. The confluency should be about 70%.
2. To prepare the DNA/liposome complex for cell transfection at RT, dilute 1 μg of bacmid DNA in 50 μL ESF921 medium (−a/a). In parallel, dilute 3 μL of ESCORT IV in 47 μL of the same medium. Combine the DNA and liposome solutions, gently flick the tube to mix, and incubate at RT for 45 min. Prepare a control setup without Escort IV.
3. Wash the adherent cells twice with 1 mL of ESF921 medium (−a/a) and discard the supernatant. Add 0.4 mL of fresh medium (−a/a) to each well. Slowly add the DNA/liposome complex (100 μL) dropwise to the adherent cells, ensuring even coverage across the well. Incubate the plate for 15 to 18 h at standard cell culture conditions.
4. Replace the transfection medium with 1 mL of fresh ESF921 medium (+a/a) and incubate for 4 days at standard conditions.
5. Evaluate the transfection success by EYFP fluorescence microscopy (40× objective, excitation at 513 nm, emission at 527 nm). Several clusters of fluorescent cells should be visible within one well. Otherwise, increase incubation time by 1–2 days.
6. If transfection is successful, collect the entire medium from the well (P1 virus stock) and use it to directly infect a 4 mL suspension culture of 1.5 × 10^6^ Sf9 cells·mL^−1^ in a 25 cm^2^ flask. Incubate at 27 °C and 100 r.p.m. on an orbital shaker for 4–5 days.
7. Check the EYFP fluorescence (step 5) to assess the infection efficiency. If fluorescence is not observed in all cells, continue incubation for an additional 1 or 2 days (Tips & Tricks 9). Harvest 2 × 1.8 mL of the culture medium and centrifuge at 20 000 ***g*** for 30 s (RT) in aerosol‐tight tubes to remove cell debris. Transfer the supernatant into fresh aerosol‐tight tubes for storage (P2 virus stock) (Tips & Tricks 10).
8. For virus stock titration using the endpoint dilution method (TCID_50_·mL^−1^ determination) [38], plate 3 × 10^4^ High Five cells per well in a 96‐well plate with 180 μL ESF921 medium (+a/a). Use 6 wells per row for each virus stock, and 8 rows for titration. Adhere the cells for 60 min.
9. Prepare 150 μL of a 1 : 10 dilution of the virus stock in ESF921 medium (+a/a). Add 20 μL of the diluted stock to the 6 wells of the first row without mixing. Use a multi‐pipette to mix the wells of the first row simultaneously and transfer 20 μL of the supernatant into the 6 wells of the second row. Repeat this process down all 8 rows, changing pipette tips between each row and discarding 20 μL from the last row. Incubate the plate for 4 days at standard conditions.
10. Check the EYFP fluorescence (step 5). Wells with at least two fluorescent cells are considered positive for infection. Determine the TCID_50_·mL^−1^ as described below (Tips & Tricks 11).

### Intracellular protein crystallization

**Fig. 5:**
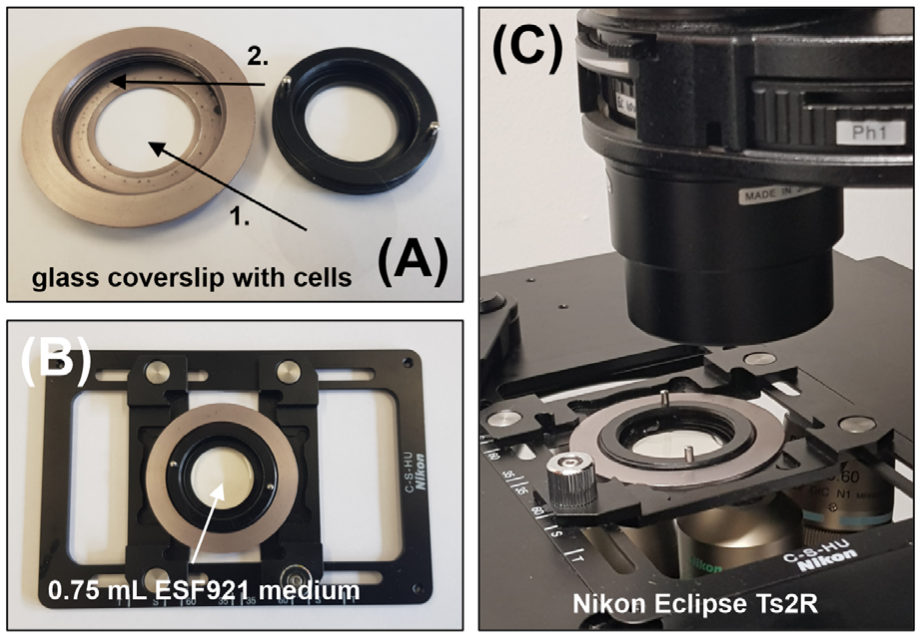
Custom‐made coverslip‐holding device. (A) For crystal‐containing cell screening using an 100 × oil immersion objective and DIC, the glass coverslip with the adherent cells is transferred from the well of a 6‐well plate onto the lower metal ring structure (1). The upper plastic part is carefully screwed in (2) to seal the upper chamber. (B) The device is placed into a sample support, and 0.75 mL of ESF921 medium (+a/a) is pipetted into the chamber to keep the cells alive during light microscopy. (C) The assembled device is placed on the *x*–*y* table of the Nikon Eclipse Ts2R microscope.

1. Plate 0.45 × 10^6^ High Five cells in 2 mL ESF921 medium (+a/a) in a well of a 6‐well plate that contains a glass coverslip. Adhere the cells for 20 min.
2. Infect the cells with the rBV at a multiplicity of infection (MOI) of 1 (Tips & Tricks 12). Add the virus stock dropwise in a spiral pattern to ensure even distribution across the well, then gently cross‐shake the plate to mix. Incubate at standard conditions for 4–5 days.
3. Screen the wells for protein crystal growth using differential interference contrast (DIC) light microscopy at 40× objective/numerical aperture (NA) 0.60, e.g., using a Nikon Ti2‐Eclipse microscope equipped with a Nikon Qi2 camera and the NIS‐Elements br software (Fig. 3).
4. Image each well with diascopic mode to observe cell survival, confluency, and the growth of defined structures with distinct shapes and sizes (maximum light intensity, exposure time: 10–50 ms, resolution: 14‐bit 4908 × 3264) (Fig. S1).
5. Switch to epifluorescence mode to assess infection efficiency via excitation of the EYFP encoded in the bacmid. Adjust the filter turret to select the green fluorescence LED light and focus by adjusting the nosepiece (exposure time: 1–10 s).
6. For structure detection at 100× objective, carefully lift the glass coverslip containing the adherent cells with a 90° bent needle and transfer it with a plastic tweezer into a holding device (Fig. 5). Add 0.5–0.75 mL of ESF921 medium and analyze the cells in detail at 100× magnification (NA 1.30 oil immersion objective) (Tips & Tricks 13).

### Serial X‐ray diffraction data collection

1. Infect 0.45 × 10^6^ High Five cells in 2 mL ESF921 medium (+a/a) in a well of a 6‐well plate with an rBV at an MOI of 1 and incubate for 4–6 days at 27 °C.
2. Discard 1 mL of the medium. Gently remove the crystal‐containing High Five cells from the well by sloughing and transfer the cell suspension into a sterile 1.5 mL tube. Allow the cells to settle for 30 min.
3. Adjust a clean MicroMesh mounted on a magnetic goniometer base in the optical focus of a standard upright cell culture light microscope in a continuous humid air stream (Fig. 4). Pipette 0.5 μL of the loose cell pellet onto the mesh surface. Remove the excess liquid with an extra‐fine liquid wick from the back of the mesh to form a thin cell monolayer (Tips & Tricks 14).
4. For cryoprotection, pipette 0.5 μL of a 40% PEG200 solution in ESF921 medium onto the cells on the mesh and remove the excess liquid using a liquid wick. Immediately plunge freeze the loaded MicroMesh in liquid nitrogen and store at −196 °C until diffraction data collection (Tips & Tricks 15).
5. For serial X‐ray diffraction data collection at a synchrotron radiation source (e.g., PETRA III at DESY in Hamburg, Germany), mount the cryo‐cooled MicroMeshes at 100 K in a gaseous nitrogen stream on the goniostat at the diffractometer, either manually or automatically. Perform a serial helical grid scan strategy for data collection (Tips & Tricks 16). Using an X‐ray beam of 7 × 3 μm with a flux of 1.7 × 10^13^ photons·s^−1^, each mesh position can be irradiated for 20 ms without detectable radiation damage. Other suitable synchrotron facilities for microcrystal X‐ray diffraction are listed in [35] (Tips & Tricks 17).
6. Process the collected diffraction patterns, e.g., by applying the crystfel software suite [36] for snapshot serial crystallography, as described in [26].

## Tips & Tricks

1. Design the target gene‐specific PCR primers without start/stop codons (encoded in the pFB1 plasmid). For restriction cloning, the fwd primer has to encode a *Kpn*I restriction site and the rev primer a *Nhe*I site at the 5′ end (Fig. 2B).
2. The optimal annealing temperature can be calculated, e.g. using https://tmcalculator.neb.com/#!/main (Q5 High‐Fidelity DNA Polymerase, 200 nm primer concentration).
3. Run an analytical agarose gel electrophoresis after PCR amplification of the target gene. If no unspecific DNA bands are detected, proceed with PCR Clean‐up instead of Gel Clean‐up to decrease the loss of the PCR product during purification (see NucleoSpin® Gel and PCR Clean‐up Kit's guidelines).
4. Cellular compartment screening should be strategized based on the post‐translational modifications of the target protein and on its native compartment, which represents the most successful environment for intracellular crystallization in insect cells so far [26]. For cytosolic proteins, gene cloning into pFB1 cyto, pFB1 PTS1, and pFB1 NLS is recommended for first crystallization tests, while genes encoding glycosylated or secreted proteins should be initially cloned into pFB1 SS and SS‐R.
5. Store the remaining ligation reaction at −20 °C. If the transformation is unsuccessful, perform the transformation again with 4–6 μL of the stored ligation reaction to increase the transformation efficiency.
6. Optionally, confirmation of successful ligation can be performed by digesting 1 μg of plasmid DNA with 1 μL FD *Kpn*I and 1 μL FD *Nhe*I in a 20 μL volume for 45 min at 37 °C. However, *Bam*HI and *Hind*III have not been used for restriction cloning and cleave the pFB1 plasmid upstream of the start and downstream of the stop codon (Fig. 2A), resulting in slightly differing DNA band sizes compared to that resulting from *Kpn*I and *Nhe*I digestion.
7. Basic insect cell culture maintenance techniques and the guidelines of the Sf9 and High Five insect cell manual (https://www.fishersci.de/de/de/home.html) should be followed. All steps must be carried out carefully and precisely under sterile conditions.
8. If only small amounts of cells are required, subculturing can also be performed in 25 cm^2^ flasks using a maximum culture volume of 7 mL. However, High Five cells show increased signs of stress when cultured in T25 flasks, affecting cell viability.
9. Ensure the 4‐day incubation time is not exceeded if it is required to minimize the amount of defective viral particles.
10. Virus stocks can be stored in the dark at 4 °C for up to 1 year. The addition of 2.5% sucrose (w/v) will improve the activity of the virions. For longer storage, plunge‐freeze the sucrose‐containing stocks in liquid nitrogen and store at −80 °C or −196 °C.
11. The TCID_50_·mL^−1^ can be calculated using the Excel sheet provided in the supplementary material of [26]. Fill in ‘1’ for a positive and ‘0’ for a negative well detected in the 96‐well plate in the upper scoring area, and your specific experiment parameters in lines 15 to 18. The TCID_50_·mL^−1^ is automatically calculated and presented in line 27. If the TCID_50_·mL^−1^ of the P2 stock is below 1 × 10^7^·mL^−1^, produce a P3 stock by repeating steps 6 and 7. Infect the suspension culture with at least an MOI of 1 using the P2 stock.
12. Calculate the rBV stock volume required to infect High Five cells with a given MOI: $V_{\text{virus}}\;\left[\mathrm{mL}\right]=\frac{\mathrm{MOI}\times \text{cell count}}{{\text{TCID}}_{50}\;{\mathrm{mL}}^{-1}\times 0.7}$.
13. As previously reported, infection with an MOI of more than 1 does not affect crystallization efficiency and crystal size for most of the successfully crystallized proteins [26]. However, the MOI can be increased up to 20 if no indications of intracellular crystals have been obtained. Ordered structures in a living cell are strong indications of a crystalline protein. Screen for needle‐, square‐, or rectangular‐shaped objects, as exemplarily shown in Fig. 3. Confocal microscopy or immunofluorescence might be an option to visualize even small ordered structures. Validate the crystalline state by TEM [7, 18, 26, 28] or by SAXS‐XRPD [32].
14. A continuous humid air stream is required to prevent immediate dehydration of the cells on the MicroMesh surface. For optimal serial diffraction data collection, a cell monolayer is required to avoid multiple crystal hits that cannot be processed. Moreover, the thickness of the cell layer correlates with the probability of icing, which strongly restricts data collection.
15. As an alternative to cell mounting via MicroMeshes, CrystalDirect™ plates (modified 96‐well vapor diffusion plates containing 25 μm ultrathin cyclic olefin copolymer (COC) film as crystallization support) [39] can be used for insect cell growth, rBV infection, crystal screening, and *in situ* X‐ray diffraction data collection [26]. Sterilize the plates with UV light for 40 min and incubate with 75 μL per well of a 0.2 mg·mL^−1^ poly‐d‐lysine solution for 1 h at RT. Wash the wells twice with 100 μL PBS per well. Plate 1 × 10^4^ High Five cells per well in 50 μL ESF921 medium (+a/a) and adhere the cells for 30 min. Infect each well with a rBV at an MOI of 1 by exchanging the medium with the virus stock diluted in 50 μL ESF921 medium (+a/a) supplemented with 25% FBS. Fill the outermost row of wells with 100 μL water and cover the plate with the lid to avoid drying the wells, and incubate at 27 °C for 4 days. For X‐ray diffraction, fill the reservoirs with 50 μL of water and completely remove the medium from the adherent cells. Immediately seal the plate to be airtight using ultrathin sealing film on top and directly mount upright at the appropriate sample holder of the MD3 diffractometer at the P14 beamline at PETRAIII (DESY, Hamburg).
16. For that, rasters were predefined across the mesh surface with a defined spacing between data collection points. During diffraction data collection in each vertical line, the goniostat was rotated and translated continuously. At the end of each line, the mesh was translated to the side, and the rotation and translation direction was inverted [23, 26, 34].
17. If the volume of the obtained intracellular crystals is too small to diffract synchrotron radiation at sufficient resolution (usually less than 5 μm^3^, depending on the protein, on the beam size, and on the photon flux), the SFX approach at an XFEL can be used for X‐ray diffraction data collection. If sufficiently stable, crystals can be isolated after cell lysis, concentrated, and injected into the pulsed XFEL beam using liquid or viscous jet techniques. Details for sample preparation and SFX data collection have been previously reported [7, 18, 30]. If crystal isolation impairs the crystal integrity, the entire crystal‐containing cells can be loaded on a fixed target (silicon chip) for SFX data collection following the previously described protocol [15].

## Conflict of interest

The authors declare no conflict of interest.

## Author contributions

RS and LR conceived and designed the protocol. Supervised by LR, RS, JB, JML‐R, NE, FAS, AMF, HM, and LW performed gene cloning, insect cell culture, rBV generation, as well as intracellular crystallization experiments; Crystal‐containing cell samples were prepared and characterized by RS, JB, JML‐R, NE, FAS, AMF, HM, and LW under the supervision of LR; X‐ray diffraction experiments were carried out by RS, JB, JML‐R, NE, FAS, AMF, HM, and LW and LR. The manuscript was prepared by RS, NE, FAS, AMF, HM, LW, and LR with discussions and improvements from all authors.

## Supporting information


**Fig. S1.** Microscopic image acquisition using the NIS‐Elements br software.

**File S1.** Plasmid pFB1 v2 cyto DNA sequence (pfb1‐v2‐cyto.fasta).
**File S2.** Plasmid pFB1 v2 MTS1 DNA sequence (pfb1‐v2‐mts1.fasta).
**File S3.** Plasmid pFB1 v2 MTS2 DNA sequence (pfb1‐v2‐mts2.fasta).
**File S4.** Plasmid pFB1 v2 NLS DNA sequence (pfb1‐v2‐nls.fasta).
**File S5.** Plasmid pFB1 v2 PTS1 DNA sequence (pfb1‐v2‐pts1.fasta).
**File S6.** Plasmid pFB1 v2 SS DNA sequence (pfb1‐v2‐ss.fasta).
**File S7.** Plasmid pFB1 v2 SS‐R DNA sequence (pfb1‐v2‐ss‐r.fasta).
**File S8.** Custom‐made coverslip‐holding device: Mechanical design file (FreeCAD) for the metal ring structure (coverslip‐holding‐devive_metal.FCStd).
**File S9.** Custom‐made coverslip‐holding device: Mechanical design file (FreeCAD) for the plastic inset (coverslip‐holding‐device_inset.FCStd).
**File S10.** Custom‐made coverslip‐holding device: .step file for production of the metal ring using a CNC milling machine (Coverslip‐holding‐device_metal.step).
**File S11.** Custom‐made coverslip‐holding device: .stl file for production of the plastic inset using a 3D printer (Coverslip‐holding‐device_insert.stl).

## Data Availability

Datasets presented in the figures and the experimental setups are available upon request. All modified pFB1 plasmids encoding the different intracellular translocation signals or tags are available upon request for nonprofit research organizations.
