# Supplementary material for: Intracellular protein crystallization in living insect cells
Source: FEBS Open Bio. 2025 Mar 28;15(4):551–62. doi: 10.1002/2211-5463.70020 (PMC11961387; doi:10.1002/2211-5463.70020)
Supplement: Supplementary file 1 — Fig. S1. Microscopic image acquisition using the NIS‐Elements br software. [file FEB4-15-551-s001.docx]

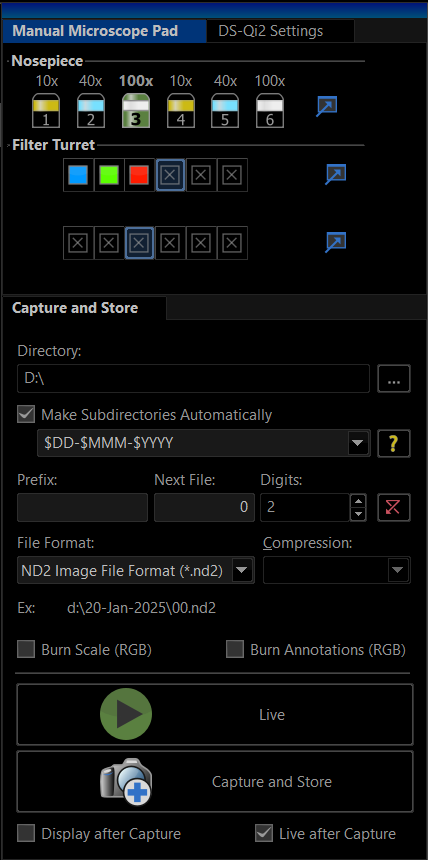

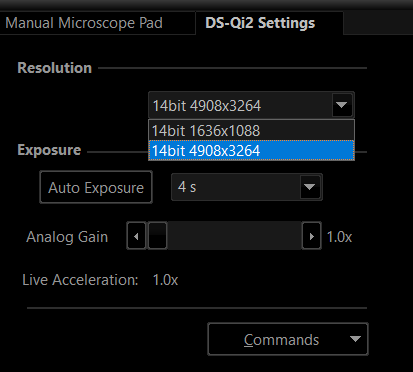

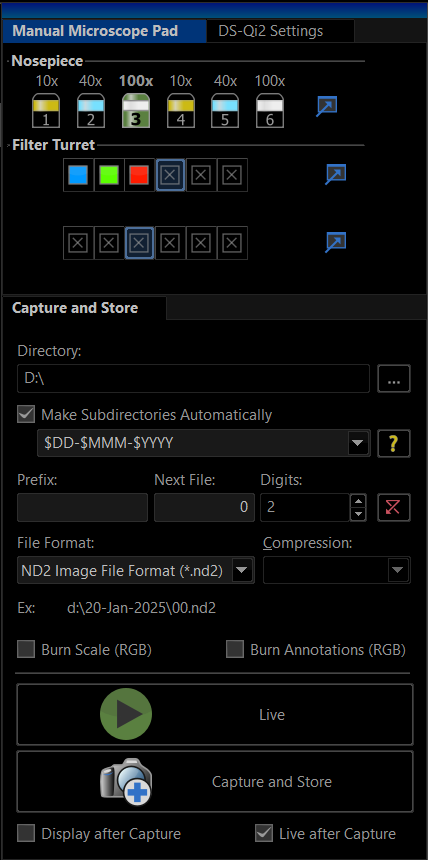


**B)**

**A)**

**C)**

**Supplementary Fig. 1**. Microscopic image acquisition using the NIS-Elements BR software. (A) Select the correct nosepiece and the filter turret under “Manual Microscope Pad”. (B) Next, switch to “DS-Qi2 Settings” to adjust the resolution (14-bit 4908x3264) and the auto exposure accordingly (10-50 ms for DIC light microscopy/1-10 s for EYFP fluorescence microscopy). (C) Subsequently, under “Capture and Store”, choose the appropriate directory, the prefix of the specimen, and the file format (*.nd2). Click on “Live” for data acquisition and “Capture and Store” to save the images.
